# Three-year outcomes of vitrectomy combined with intraoperative dexamethasone implantation for non-tractional refractory diabetic macular edema

**DOI:** 10.1038/s41598-020-80350-w

**Published:** 2021-01-14

**Authors:** Sungsoon Hwang, Se Woong Kang, Kyung Tae Kim, Hoon Noh, Sang Jin Kim

**Affiliations:** 1 Department of Ophthalmology, Samsung Medical Center, Sungkyunkwan University School of Medicine, #81 Irwon-ro, Gangnam-gu, Seoul, 06351 Republic of Korea; 2 Department of Ophthalmology, Gangneung Asan Hospital, College of Medicine, University of Ulsan, Ulsan, Republic of Korea

**Keywords:** Retinal diseases, Diabetes complications

## Abstract

This retrospective, consecutive interventional study investigated the long-term clinical outcomes of combined vitrectomy with intraoperative dexamethasone implants for non-tractional refractory diabetic macular edema (DME). The study included 43 eyes from 39 participants with DME that had continued for more than 6 months despite repeated non-surgical treatment. Postoperative changes in best-corrected visual acuity (BCVA) and central macular thickness (CMT) were evaluated over 3 years. A Kaplan–Meier curve was obtained for any additional non-surgical treatment, and the average number of non-surgical treatments required for DME before and after surgery was compared. Other postsurgical complications were also investigated. The logMAR BCVA improved from 0.526 ± 0.417 (20/67) preoperatively to 0.294 ± 0.374 (20/39) 3 years postoperatively (*p* < 0.001, generalized estimating equation). The CMT improved from 478 ± 122 μm preoperatively to 314 ± 90 μm 3 years postoperatively (*p* < 0.001, generalized estimating equation). Additional non-surgical treatment was not required for 29 (67%) eyes. The average number of annual non-surgical treatments decreased from 5.04 times preoperatively to 0.34 times postoperatively. Seventeen (40%) eyes developed temporary ocular hypertension after surgery, which normalized after antihypertensive eye drop instillation. In conclusion, vitrectomy combined with intraoperative dexamethasone implantation provides satisfactory long-term clinical outcomes for non-tractional refractory DME while reducing the number of intraocular injections for DME.

## Introduction

Diabetic macular edema (DME) is a major cause of visual impairment in patients with diabetes^1^. It is characterized by the accumulation of fluid and/or protein within the retinal layer of the macula caused by microvascular compromise, Muller cell dysfunction, and blood-retinal barrier breakdown^2^. Non-surgical treatments, including macular laser photocoagulation^3^, intravitreal injections of either anti-vascular endothelial growth factor (VEGF) agents^4,5^ or corticosteroids^6,7^, and posterior sub-tenon injection of corticosteroids^8,9^, are considered the main treatment methods for DME. However, in cases where DME does not respond successfully to these non-surgical treatments, vitrectomy can be considered an alternative therapeutic approach^10–12^.

Conventionally, intravitreal triamcinolone has been used in combination with vitrectomy since it has proven to be effective in preventing various postsurgical complications, such as macular edema^13^. However, a limitation of this intervention is the increased clearance of triamcinolone in vitrectomized eyes, approximately six times faster than that in non-vitrectomized eyes^14^.

Ozurdex (Allergan, Irvine, CA, USA), a dexamethasone intravitreal implant, is a biodegradable device that releases dexamethasone for up to 6 months in both vitrectomized and non-vitrectomized eyes^15,16^. Numerous studies have reported that Ozurdex is a safe and effective management modality for DME^7,17,18^ and can delay the progression of diabetic retinopathy^19–21^. Considering the clearance issue of triamcinolone in vitrectomized eyes, Ozurdex is a good alternative for intravitreal triamcinolone in combination with vitrectomy for DME resistant to other treatments.

Previously, there have been a few studies assessing the clinical outcomes of vitrectomy combined with intraoperative dexamethasone implantation for DME^22–24^. However, these studies only reported short-term clinical results of up to a maximum of 1 year. Therefore, we investigated the long-term clinical outcomes of vitrectomy combined with intravitreal dexamethasone implantation for non-tractional refractory DME.

## Methods

### Setting

This was a retrospective interventional study of consecutive patients with non-tractional refractory DME who were treated with vitrectomy combined with intravitreal dexamethasone implantation and were followed up for at least 3 years after surgery. The study adhered to the tenets of the Declaration of Helsinki and was approved by the Institutional Review Board of Samsung Medical Center, Seoul, Republic of Korea (IRB number 2020-02-124). The board waived the requirement for informed consent owing to the retrospective design of the study.

### Subjects

The electronic medical records of all consecutive patients who were diagnosed with non-tractional refractory DME with central macular thickness (CMT) greater than 300 μm at Samsung Medical Center between January 2015 and March 2017 were retrospectively reviewed. Non-tractional refractory DME was defined as biomicroscopically, angiographically, and tomographically confirmed DME that had continued for more than 6 months despite repeated non-surgical treatments (including macular laser photocoagulation, posterior sub-tenon corticosteroid injection, intravitreal corticosteroid injection, and intravitreal injection of anti-VEGF agents) in the absence of any evidence of traction force (e.g., vitreomacular traction or epiretinal membrane) on optical coherence tomography (OCT). Subjects with active proliferative diabetic retinopathy requiring surgical management, those with prior history of vitreoretinal surgery, those with evidence of any other past or concomitant retinal diseases that might affect visual acuity or macular microstructure, and those who were lost to follow-up before 3 years postoperatively were excluded from the study. Among the 52 eyes with non-tractional refractory DME that met the study inclusion criteria, 9 eyes were excluded owing to the following reasons: active vitreous hemorrhage at the time of surgery (2 eyes), history of vitreoretinal surgery (1 eye), follow-up period of less than 3 years (6 eyes). Finally, 43 eyes of 39 patients were included in the study.

### Preoperative examination

All subjects underwent thorough preoperative ocular examination, including best-corrected visual acuity (BCVA) measurement using a Snellen chart, manifest refraction, applanation tonometry, slit-lamp biomicroscopy, and dilated fundus examination. Wide-field fluorescein angiography was routinely performed preoperatively to evaluate the severity of diabetic retinopathy and the extent of the non-perfusion area, and spectral domain OCT (Spectralis HRA-OCT; Heidelberg Engineering, Heidelberg, Germany) was performed to evaluate any vitreomacular interface abnormalities and measure the CMT.

### Surgical technique

Standard transconjunctival sutureless pars plana vitrectomy was performed by a single surgeon (SWK) using a Constellation (Alcon Laboratories, Fort Worth, TX, USA) or Associate (Dutch Ophthalmic Research Center, Zuidland, the Netherlands) 23-gauge vitrectomy system under retrobulbar anesthesia. The internal limiting membrane (ILM) was removed with a radius of around 2-disc diameters from the fovea using intraocular forceps with the assistance of indocyanine green staining in some cases. Panretinal endolaser photocoagulation was performed concomitantly for patients with extensive retinal capillary dropout or with apparent high-risk characteristics. At the end of the operation, a dexamethasone implant was placed into the vitreous cavity through the 23-gauge vitrectomy port. Patients were advised to maintain a sitting position for a few hours to position the dexamethasone implant at the inferior periphery. Combined cataract surgery was performed in patients aged over 50 years to pretreat post-vitrectomy lens opacity.

### Postoperative examination and management

Patients were routinely followed up at 1, 3, 6, 12, 24, and 36 months postoperatively in cases in which DME resolved and stabilized without further treatment. At every visit, patients underwent BCVA measurement, slit-lamp biomicroscopy, applanation tonometry, dilated fundus examination, and OCT. If macular edema did not respond to the surgery until postoperative 3 months or recurred/aggravated after resolution, non-surgical treatments were restarted and followed up according to an individual’s ocular condition. Patients who had intraocular pressure (IOP) greater than 21 mmHg were prescribed IOP-lowering eye drops, as recommended by glaucoma specialists, and managed accordingly.

### Statistical analyses

We collected clinical data on BCVA, CMT, IOP, glaucoma medication usage, additional non-surgical treatment for DME after surgery, and other unexpected ocular complications from the preoperative period to 3 years after surgery. Improvement of BCVA (converted to a logarithm of the minimal angle of resolution [logMAR] scale) and CMT were assessed at postoperative 3, 6, 12, 24, and 36 months using a generalized estimating equation accounting for the correlation of paired eyes. We obtained a Kaplan–Meier curve of additional treatment requirement for DME after surgery and compared the average number of non-surgical treatments required for DME before and after surgery using a generalized estimating equation model. All statistical analyses were performed using Statistical Package for the Social Sciences (SPSS) software version 23.0 (SPSS, Inc., Chicago, IL, USA). *p* values were two sided and considered statistically significant for values less than 0.05.

## Results

### Baseline demographics

The baseline characteristics of 43 eyes from 39 patients with non-tractional refractory DME are presented in Table 1. The mean (± standard deviation) age, number of prior injections of anti-VEGF for DME, and time period of non-surgical treatment before surgery were 57.8 ± 8.2 years, 6.25 ± 7.12, and 17.0 ± 14.2 months, respectively. Twelve eyes had a history of cataract surgery, and 24 eyes underwent cataract surgery combined with vitrectomy. Four eyes were treated with IOP-lowering medication before surgery.

**Table 1:** Baseline demographics and clinical characteristics of subjects with diabetic macular edema refractory to prior treatments.

Variables	Values
Number of patients (eyes)	39 (43)
Age, years, mean ± SD	57.8 ± 8.2
Sex, male: female	26:13
Type of DM, type 1:type 2	0:39
Duration of diabetes, years, mean ± SD	13.3 ± 8.6
Presence of hypertension, No. (%)	19/39 (48.7)
Presence of dyslipidemia, No. (%)	7/39 (17.9)
Severity of diabetic retinopathy, NPDR:PDR	11:32
Lens type, phakic:pseudophakic	31:12
Combined cataract surgery with vitrectomy, No. (%)	24/31 (77.4)
**Treatment modalities used prior to the surgery**	
Intravitreal anti-VEGF, No. (%)	42/43 (97.7)
Number of treatment session, mean ± SD (range)	6.5 ± 7.1 (1–32)
Intravitreal dexamethasone, No. (%)	5/43 (11.6)
Number of treatment session, mean ± SD (range)	1.6 ± 0.9 (1–3)
Intravitreal triamcinolone, No. (%)	9/43 (20.9)
Number of treatment session, mean ± SD (range)	2.7 ± 2.8 (1–10)
Posterior sub-tenon triamcinolone, No. (%)	4/43 (9.3)
Number of treatment session, mean ± SD (range)	3.3 ± 4.5 (1–10)
Macular laser photocoagulation, No. (%)	9/43 (20.9)
Number of treatment session, mean ± SD (range)	1.9 ± 1.5 (1–5)
Duration of prior treatment for DME, months, mean ± SD	17.0 ± 14.2
Time between the last treatment and surgery, months, mean ± SD	2.83 ± 1.43
Preoperative BCVA, logMAR, mean ± SD	0.526 ± 0.417
Preoperative CMT, μm, mean ± SD	478 ± 122
Preoperative IOP, mmHg, mean ± SD	17.0 ± 2.8
On glaucoma medication (preoperatively), No. (%)	4/43

SD, standard deviation; DM, diabetes mellitus; NPDR, non-proliferative diabetic retinopathy; PDR, proliferative diabetic retinopathy; DME, diabetic macular edema; VEGF, vascular endothelial growth factor; BCVA, best-corrected visual acuity; logMAR, logarithm of the minimal angle of resolution; CMT, central macular thickness; IOP, intraocular pressure.

### Clinical outcomes

**Figure 1:**
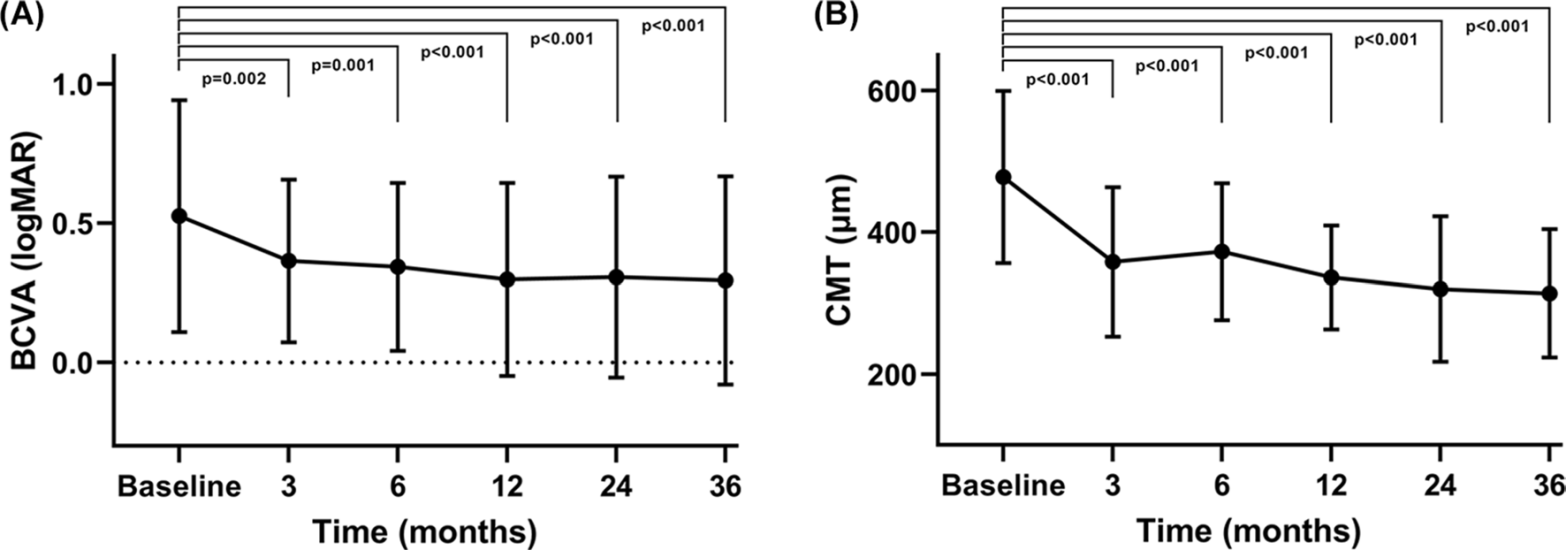
Changes in the mean and standard deviation of the logarithm of the minimal angle of resolution (logMAR) best-corrected visual acuity (BCVA) and central macular thickness (CMT) at 3, 6, 12, 24, and 36 months after vitrectomy combined with intraoperative dexamethasone implantation for non-tractional refractory diabetic macular edema. Statistical significance was determined using a generalized estimating equation model accounting for the correlation structure of paired eyes. (**A**) The logMAR BCVA significantly improved postoperatively compared to the baseline during follow-up. (**B**) The CMT was significantly thinner postoperatively than preoperatively. The average CMT at 6 months was slightly thicker than that at 3 months, but the difference was not statistically significant (*p* = 0.258).

The logMAR BCVA was 0.526 ± 0.417 preoperatively, which gradually improved to 0.294 ± 0.374 at postoperative 3 years (*p* < 0.001). The CMT was 478 ± 122 μm before the surgery, which improved to 314 ± 90 μm 3 years after surgery (*p* < 0.001). The improvements were significant throughout the follow-up period. Figure 1 demonstrates the improvement in BCVA and CMT after surgery.

**Figure 2:**
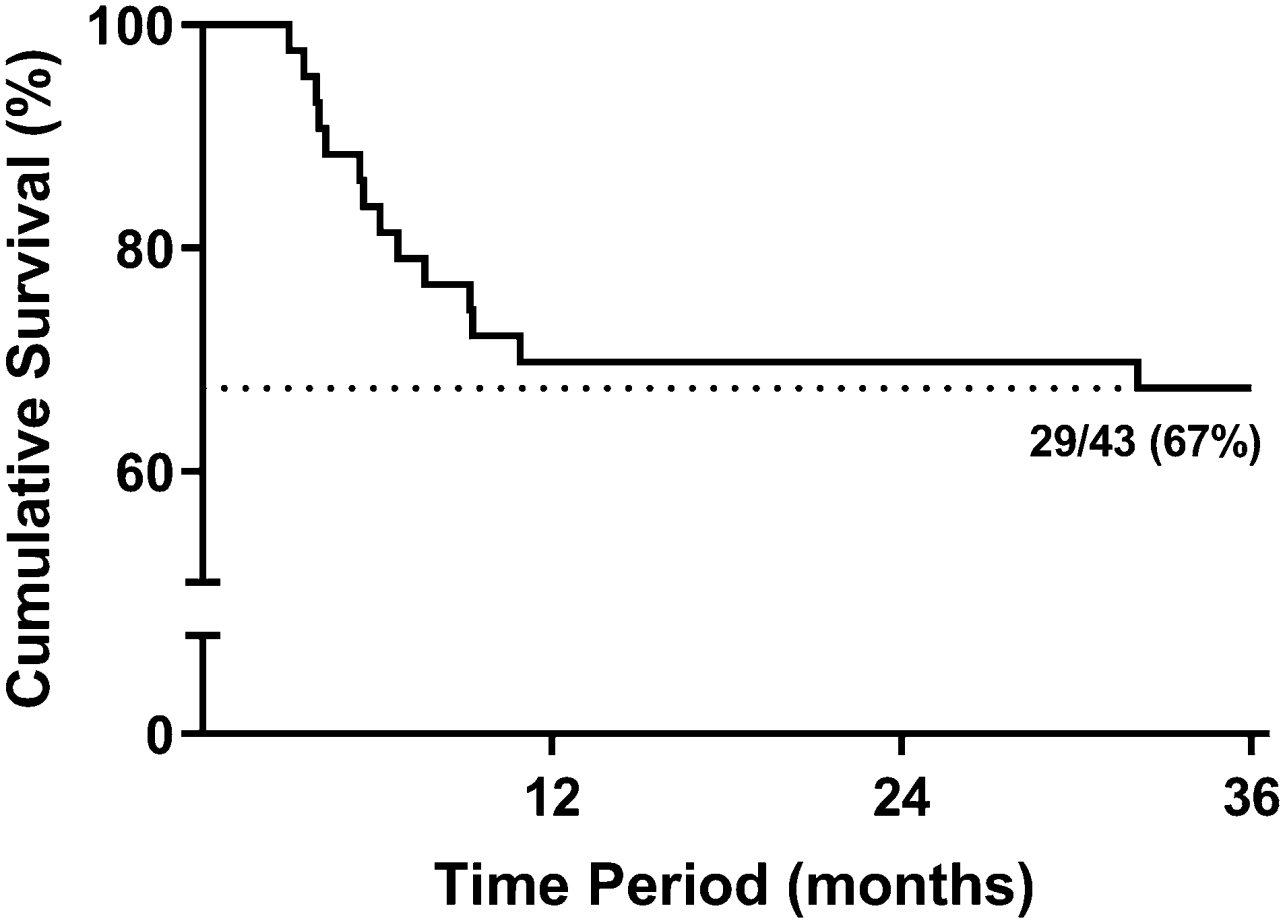
The Kaplan–Meier curve for additional nonsurgical treatment requirement after vitrectomy combined with intraoperative dexamethasone implantation for non-tractional refractory diabetic macular edema. Twenty-nine of 43 (67%) eyes maintained the resolved state for 3 years, and 14 of 43 (33%) eyes required further treatment for diabetic macular edema. Ten of 43 (23%) eyes experienced temporary resolution of macular edema between 1 and 6 months postoperatively, but the macular edema recurred during the follow-up period and underwent additional treatment. Four (9%) subjects did not achieve resolution of macular edema and underwent additional treatment after 3 months postoperatively.

Twenty-nine of the 43 (67%) eyes responded well to the surgery and maintained the resolved state for 3 years. Fourteen (33%) eyes required additional non-surgical treatment for DME during the study period; 13 eyes required additional treatment within 1 year, and one eye received additional treatment at 32 months postoperatively. Figure 2 shows the Kaplan–Meier curve of additional treatment requirements for DME.

Table 2 presents the average annual number of non-surgical treatments performed before and after surgery. The number of intravitreal injection significantly decreased after surgery (*p* < 0.001), even in the subgroup analysis performed with patients who required additional treatment for more than once after surgery (*p* = 0.001).

**Table 2:** Number of treatments for diabetic macular edema per year before and after vitrectomy combined with intraoperative dexamethasone implantation.

	First visit to operation	Operation to postoperative 3 years	*p *value*
**All subjects (43 eyes)**			
Overall intravitreal injection	4.99 ± 1.82 (2.0–9.0)	0.20 ± 0.48 (0.0–2.7)	< 0.001
Anti-VEGF	4.42 ± 1.92 (0.0–9.0)	0.09 ± 0.43 (0.0–2.7)	< 0.001
Dexamethasone	0.25 ± 0.89 (0.0–4.5)	0.10 ± 0.20 (0.0–0.7)	0.280
Triamcinolone	0.33 ± 0.87 (0.0–5.1)	0.01 ± 0.05 (0.0–0.3)	0.042
Posterior sub-tenon triamcinolone	0.14 ± 0.50 (0.0–2.8)	0.04 ± 0.11 (0.0–0.3)	0.224
Laser photocoagulation	0.29 ± 0.69 (0.0–2.8)	0.17 ± 0.34 (0.0–1.3)	0.299
**Subjects who required additional treatment after surgery (14 eyes)**			
Overall intravitreal injection	5.12 ± 1.92 (2.0–8.3)	0.57 ± 0.68 (0.0–2.7)	0.001
Anti-VEGF	4.49 ± 2.17 (0.0–7.8)	0.26 ± 0.71 (0.0–2.7)	0.001
Dexamethasone	0.47 ± 1.40 (0.0–5.1)	0.29 ± 0.26 (0.0–0.7)	0.624
Triamcinolone	0.15 ± 0.32 (0.0–1.0)	0.02 ± 0.09 (0.0–0.3)	0.053
Posterior sub-tenon triamcinolone	0.13 ± 0.34 (0.0–1.1)	0.12 ± 0.17 (0.0–0.3)	0.880
Laser photocoagulation	0.56 ± 0.71 (0.0–2.3)	0.45 ± 0.45 (0.0–1.3)	0.654

VEGF, vascular endothelial growth factor.

Variables are described as mean ± standard deviation (range).

*p values were calculated using a generalized estimating equation accounting correlation structure for paired eyes.

### Adverse events

Cases of postoperative leakage/hypotony, new retinal tears/breaks, anterior migration of the dexamethasone implant, or endophthalmitis were not observed. Seventeen (40%) eyes experienced a temporary increase in IOP (> 21 mmHg) after surgery, but the IOP normalized after using IOP-lowering eye drops. None of the subjects required filtering surgery within 3 years of vitrectomy. Of the seven eyes remaining phakic after vitrectomy, five eyes eventually underwent cataract surgery 16.4 ± 11.5 months after vitrectomy. One eye developed intraocular lens dislocation at 26 months postoperatively and underwent intraocular lens scleral fixation. No other complications were observed during the follow-up period.

## Discussion

The present study elucidated that vitrectomy combined with intraoperative dexamethasone implantation can be an effective treatment for non-tractional refractory DME. The strong point of the current study is the confirmation of the long-term stability and efficacy of vitrectomy combined with intraoperative dexamethasone implantation for refractory DME. Additionally, the study also suggested that surgery may reduce the number of intravitreal injections required for managing DME.

Currently, the standard treatment method for DME is an intravitreal anti-VEGF injection or corticosteroid injection^4–7^. Some studies have suggested good long-term functional and anatomical outcomes of primary vitrectomy for treatment-naïve DME^25^, but most clinicians do not perform vitrectomy initially unless there is significant vitreomacular traction^26^. Anti-VEGF injections and corticosteroid injections often require frequent and continuous treatment sessions and not all patients respond successfully to the treatment^27^. This is a commonly encountered dilemma for ophthalmologists in clinical practice. In this situation, vitrectomy can be an alternative treatment. Several previous studies have reported the efficacy of vitrectomy for treatment-refractory DME^10–12,28–30^. The studies agree that vitrectomy helps reduce macular thickness in refractory DME, but improvement of visual acuity after vitrectomy has been controversial. Several long-term studies have suggested that vitrectomy for refractory DME is beneficial in both reducing CMT and improving visual acuity^10–12,29–31^. In contrast, in a large prospective study from the Diabetic Retinopathy Clinical Research Network (DRCR.net), vitrectomy was beneficial for reducing CMT; however, its efficacy for improving visual acuity was limited^28^. In addition to what has been investigated thus far, our study provides additional data on both anatomically and functionally favorable long-term results of vitrectomy combined with dexamethasone implantation for refractory DME. The more favorable visual acuity outcome of our study compared to that of DRCR.net may be attributable to the baseline characteristics of the patients^28^. Compared to DRCR.net, our study comprised only patients with type 2 diabetes who were significantly younger at the time of surgery and in whom the mean duration of diabetes was approximately 7 years shorter^28^. Additionally, all patients underwent ILM peeling and Ozurdex injection during surgery in our study, whereas only 64% underwent ILM peeling and no patient received a corticosteroid injection in DRCR.net^28^. Considering that ILM peeling may produce a more favorable visual outcome and the intraoperative use of corticosteroids plays a beneficial role after vitrectomy^11,13,32^, the difference in the detailed method of surgery (ILM peeling and intraoperative Ozurdex implant) may have produced better visual outcomes in our study than in DRCR.net.

Vitrectomy is known to treat macular edema, possibly through the following mechanisms: elimination of possible sources of traction on the macular area^26^, improvement of transvitreal oxygen delivery to the retina^33^, and removal of condensed chemical mediators that worsen the retinal vascular permeability^34^. In addition to vitrectomy, concurrent ILM peeling could also completely remove the source of traction and may facilitate the penetration of drugs into the retinal tissue^24^. In contrast, dexamethasone relieves DME in different ways. Dexamethasone blocks the production of VEGF and inflammatory mediators in the retina, inhibits macrophage and leukocyte adhesion/transmigration, and enhances the tight junctions of the blood-retinal barrier^35,36^. Combining these different mechanisms, vitrectomy with intraoperative dexamethasone implants is expected to produce a synergistic effect, provide more effective treatment, and yield favorable clinical outcomes.

There have been some reports regarding vitrectomy combined with intraoperative dexamethasone implantation for refractory DME. Lee et al.^22^ and Jung et al.^24^ reported the 1-year clinical outcomes in 18 eyes and 22 eyes, respectively. Both studies showed significant improvement in BCVA and CMT without ocular complications other than temporary IOP increase. Moreover, Pang et al. recently reported the 1-year outcome of vitrectomy combined with Ozurdex implantation for refractory macular edema secondary to diabetic retinopathy, retinal vein occlusion, and non-infectious posterior uveitis^37^. The subjects with DME in their study showed the most prominent improvement in BCVA and CMT and the longest macular edema-free period. However, the previous studies were limited by a relatively short follow-up period of 1 year or less. Therefore, they were not able to present sufficient information about the long-term efficacy, stability, and safety of vitrectomy combined with intraoperative Ozurdex implantation. Furthermore, they included both tractional and non-tractional DME in the analysis. Tractional and non-tractional DME may respond differently to vitrectomy. DME with a tractional component has better clinical outcomes than DME without a tractional component after vitrectomy^26,28^, and this is intuitively comprehensible. Therefore, it would have been better to report these two different entities separately. In contrast to the aforementioned studies, we were able to provide long-term clinical data and proved that BCVA and CMT of refractory DME improved after surgery and remained stable for 3 years. Furthermore, the present study did not include subjects with tractional components and verified favorable long-term clinical results even in non-tractional refractory DME. These are the strong points of our study.

In the present study, improvements in BCVA and CMT after surgery were significant throughout the follow-up period. However, although the mean BCVA gradually improved during follow-up, the mean CMT showed a temporary deterioration between 3 and 6 months postoperatively. The temporary increase of CMT a few months after surgery was also shown in previous studies^22–24,37^. This is consistent with the fact that the Ozurdex implant releases high concentrations of dexamethasone for approximately 2 months and subsequently releases relatively lower concentrations of dexamethasone afterward, extending the therapeutic period to 6 months^38^. A decrease in the dexamethasone concentration in the retina and vitreous fluid a few months after surgery may have caused a recurrence of macular edema in some patients and led to an increase in the average CMT. Subjects with recurring macular edema were treated accordingly, and the mean CMT improved thereafter and remained stable.

The proportion of subjects who required retreatment after surgery in the present study was similar to that reported in previous studies. Lee et al.^22^ and Jung et al.^24^ reported that 27% and 28% subjects, respectively, required additional treatment within 1 year. In our study, 33% subjects (14 eyes) required additional treatment during the study period. Most of the additional treatment resulting from the recurrence of macular edema after surgery occurs within 1 year after surgery. Among the 14 eyes that received additional treatment, 13 eyes required additional treatment within 12 months postoperatively, and only one eye required additional treatment at 32 months postoperatively. This means that eyes with DME that are successfully treated by vitrectomy combined with dexamethasone implantation and remain stable for a year are less likely to develop macular edema afterward. Additionally, the average number of intraocular injections also significantly decreased after surgery. Even the retreated subjects required fewer additional intraocular injections compared to the preoperative period. This may reduce the risk of ocular complications associated with frequent intraocular injections and may be more beneficial in terms of patient convenience and financial costs. Furthermore, prolonged treatment with anti-VEGF injections for DME can also increase the risk of cerebrovascular accidents and death^39^. Therefore, even in this anti-VEGF era, we believe that vitrectomy combined with intraoperative dexamethasone implantation could be a viable and useful option for non-tractional DME refractory to prolonged multiple injections of anti-VEGF.

Cataract formation and increase in IOP are the main concerns of both vitrectomy and intravitreal dexamethasone implantation. In our study, seven eyes were phakic after vitrectomy combined with intraoperative dexamethasone implantation. They did not undergo cataract surgery because of young age (< 50 years) at the time of vitrectomy. Five of the seven (71%) eyes later underwent cataract surgery because of the significant visual impairment caused by progression of lens opacity, and only two eyes were phakic at the end of the study period.

A temporary elevation of IOP after surgery was observed in some subjects, but it was well controlled with the use of IOP-lowering eye drops during the study period. However, a relatively large proportion of subjects (17 eyes, 40%) experienced an increase in IOP, and some of them (11 eyes, 25%) continued to use IOP-lowering eye drops until 36 months postoperatively. Additionally, one eye underwent filtering surgery for neovascular glaucoma after the study period (postoperative 47 months). Although it was reported that Ozurdex induces less IOP increase and cataract formation compared to triamcinolone^40,41^, they should not be taken lightly, and surgeons should take constant care of these complications.

This study has some strengths. It comprised a larger number of subjects than previous studies. To our knowledge, this is the first study reporting the long-term clinical outcomes of vitrectomy combined with intraoperative dexamethasone implantation for non-tractional refractory DME. However, this study has some limitations. First, due to the retrospective nature of this study, there could have been a bias in patient selection, and the preoperative and postoperative management for each individual was heterogeneous and not standardized. However, we believe that this reflects the real-world situation because different clinicians may have different treatment strategies for DME and that clinicians would try several different methods for refractory disease. Second, the results of the current study are limited by the lack of a control group. It would have been a more significant study if we were able to compare the efficacy of vitrectomy combined with intraoperative dexamethasone implantation with that of vitrectomy alone without intravitreal dexamethasone implantation or continued conventional treatment. Third, this study could not provide additional information on the predictors of postoperative clinical outcomes and when to switch from non-surgical treatment to surgical treatment for DME. Further studies addressing these issues may provide a better understanding of the benefits of vitrectomy combined with intraoperative dexamethasone implantation for non-tractional refractory DME.

In conclusion, vitrectomy combined with intravitreal dexamethasone implantation showed satisfactory long-term clinical outcomes in non-tractional refractory DME. This treatment may reduce the number of intraocular injections required for managing DME, thereby reducing ocular and systemic side effects and increasing patient comfort. Vitrectomy combined with intraoperative dexamethasone implantation could be an effective alternative treatment modality for non-tractional refractory DME.

## Data Availability

The datasets generated and/or analyzed during the current study are available from the corresponding author on reasonable request.
